# Stress Ratio and Notch Effects on the Very High Cycle Fatigue Properties of a Near-Alpha Titanium Alloy

**DOI:** 10.3390/ma11091778

**Published:** 2018-09-19

**Authors:** Kun Yang, Bin Zhong, Qi Huang, Chao He, Zhi-Yong Huang, Qingyuan Wang, Yong-Jie Liu

**Affiliations:** 1 Failure Mechanics and Engineering Disaster Prevention and Mitigation Key Laboratory of Sichuan Province, College of Architecture and Environment, Sichuan University, Chengdu 610065, China; scu_yangkun@163.com (K.Y.); hechao@cdu.edu.cn (C.H.); 2 Key Laboratory of Deep Underground Science and Engineering, Ministry of Education, Sichuan University, Chengdu 610225, China; 3 Beijing Institute of Aeronautical Materials, Aviation Industries of China, Beijing 100095, China; bin.zhong@263.net; 4 Department of Civil Engineering, Sichuan College of Architectural Technology, Deyang 618000, China; huangqiscu@gmail.com; 5 School of Architecture and Civil Engineering, Chengdu University, Chengdu 610106, China; 6 School of Aeronautics and Astronautics, Sichuan University, Chengdu 610064, China; huangzy@scu.edu.cn

**Keywords:** VHCF, multi-point surface crack initiation, notch, mean stress, fatigue strength prediction

## Abstract

Ultrasonic fatigue tests up to 10^10^ cycles were performed on a turbine engine titanium alloy (Ti-8Al-1Mo-1V) at the stress ratio (*R*) of −1 with smooth specimens and at *R* = −1, 0.1 and 0.5 with notched specimens. As a result, with increase of fatigue life, the source of reduced fatigue life caused by multi-point surface crack initiation changes from crack propagation stage to crack initiation stage in the high cycle fatigue regime. Notch effect further promotes the degeneration of high cycle and very high cycle fatigue strength at *R* > −1. The bilinear model, extended from the Goodman method, can better estimate the mean stress sensitivity of this titanium alloy. The fatigue mean stress sensitivity and fatigue-creep mean stress sensitivity of this material increased with the increase of fatigue life. The new model, based on the Murakami model, can provide more appropriate predictions for notch fatigue strength.

## 1. Introduction

Based on the difference of fatigue crack origin, Mughrabi [1] classified metallic materials as Type I or Type II. Type I materials do not contain inclusions. However, the internal non-metallic inclusions usually act as the crack origin for Type II materials [2,3], which forms a so-called “fish-eye” [4] type fatigue failure. This typical failure feature of Type II has attracted the attention of a large number of researchers. However, with higher requirements for lightweight design and corrosion resistance, research on titanium alloys (Type I materials) have been increasing in recent years [5,6,7,8,9,10,11]. The Ti-8Al-1Mo-1V titanium alloy tested in this study is used to manufacture low-pressure compressor blade for an advanced military gas turbine engine [12]. Because of the high excitation frequency of the blade (significantly in excess of 1 kHz [12]), failure occurs in the very high cycle fatigue (VHCF, beyond 10^7^ cycles) regime. The conventional fatigue limit is determined at 10^7^ cycles, which can be achieved in less than 2.8 h, far below the in-service time for blades. Thus, it is necessary to study the VHCF properties of this titanium alloy in the designing of the blade.

Bathias et al. [13] summarized that the fatigue failure of titanium alloys can occur in the VHCF regime, and the continuous declining *S*-*N* curves are detected in smooth specimens. Similar to “fish-eye” failure in Type II materials, titanium alloys can also initiate interior fatigue cracks in the VHCF regime [13,14,15,16]. Therefore, the competition between surface-induced crack initiation and interior-induced crack initiation do not necessarily lead to the appearance of a step-wise or duplex *S*-*N* curve for smooth specimens [7]. The facetted features are revealed in the crack initiation region [17,18], and it becomes more obvious as the stress ratio is increased [7,8]. However, sometimes no facet is observed at the stress ratio (*R*) of −1 [6]. Most researchers believe that the facets result from the fracture of primary α grains [7,8,17,19].

To satisfy its function, the blade is designed as possessing very complex geometry. When it operates, the sharp change of geometry will cause localized stress concentration. Stress concentration is a harmful factor for fatigue property [20]. Multi-point surface crack initiation is usually detected in a low cycle fatigue regime [14]. The stress concentration induced by notch geometry can enhance the appearance probability of this failure mode [21]. However, few reports discuss how multi-point surface crack initiation behavior affects the fatigue properties of materials. On the other hand, an investigation by Khan [22] reported that the effect of notch on fatigue property of AISI 310 stainless steel correlated with fatigue life.

The other key factor influencing the fatigue property of blades is stress ratio. It is generated by overlying the high frequency vibration load on the inertial centrifugal force, which is induced by the high-speed rotation of blades. The degeneration of VHCF properties of titanium alloys was frequently found at *R* > −1 for smooth specimen [5,7,10]. Gao [23] investigated the effects of stress ratio and notch on the high cycle fatigue (HCF) property of a steel. Two new factors were proposed by him to reflect fatigue mean stress sensitivity and stress concentration sensitivity at a specified fatigue life of 1 × 10^7^ cycles. Indeed, the coupling effects of stress ratio and notch on the HCF and VHCF properties of titanium alloys have been rarely reported.

In this paper, ultrasonic fatigue tests were performed on a turbine engine titanium alloy at *R* = −1 with smooth specimens and at *R* = −1, 0.1 and 0.5 with notched specimens for further research on the coupling effects of stress ratio and notch on the HCF and VHCF properties of this alloy. All the failure fracture surfaces were examined, and the equivalent sizes of single-point crack initiation regions were measured. The effect of multi-point crack initiation on fatigue property was discussed. A bilinear model was proposed to quantify the mean stress sensitivity of fatigue strength. Meanwhile, the new concept of fatigue-creep mean stress sensitivity factor was defined and calculated. Lastly, a newly-developed model was used to estimation of notch fatigue strength according to equivalent sizes.

## 2. Material and Experimental Procedure

**Figure 1:**
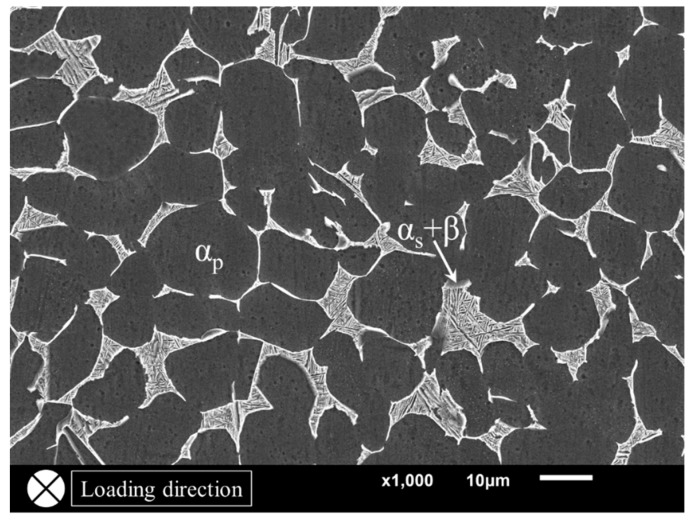
The microstructure of the Ti-8Al-1Mo-1V alloy. The testing surface corresponds to a transversal section of fatigue specimens.

A near-alpha titanium alloy (Ti-8Al-1Mo-1V) was employed in this study. The main chemical compositions (mass fraction) of this alloy were 7.79% Al, 0.98% Mo, 1.00% V and the remaining Ti. Moreover, the detected impurity elements contained Fe, C, N, H and O, and their maximal content was just up to 0.06%. The as-received materials are hot-rolled bars; no additional heat treatment was applied. Figure 1 shows the microstructure of the material along the loading direction, that is, the testing surface corresponds to a transversal section of fatigue specimens. It consisted of about 85% equiaxed primary α grain (α_p_) and the remaining 15% lamellar structure (α_s_ + β), where secondary α laths (α_s_) are randomly embedded in the β matrix. Therefore, the microstructure is of a duplex pattern.

Before conducting fatigue tests, the mechanical properties of this alloy were measured by tensile tests with two cylindrical smooth specimens of 5 mm in diameter. As a result, the yield strength of 820 MPa, the ultimate tensile strength of 928 MPa, the tensile elongation of 16% and the Young’s modulus of 113.6 GPa were determined. Meanwhile, the average hardness value of 322.2 HV1 was obtained from 10 indentation points with a stable loading of 9.8 N for 20 s.

**Figure 2:**
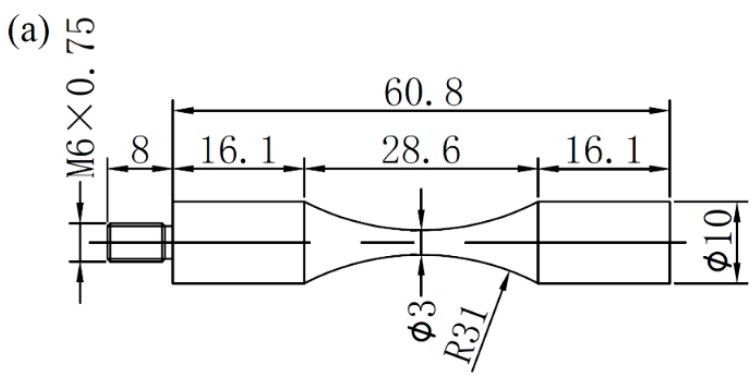

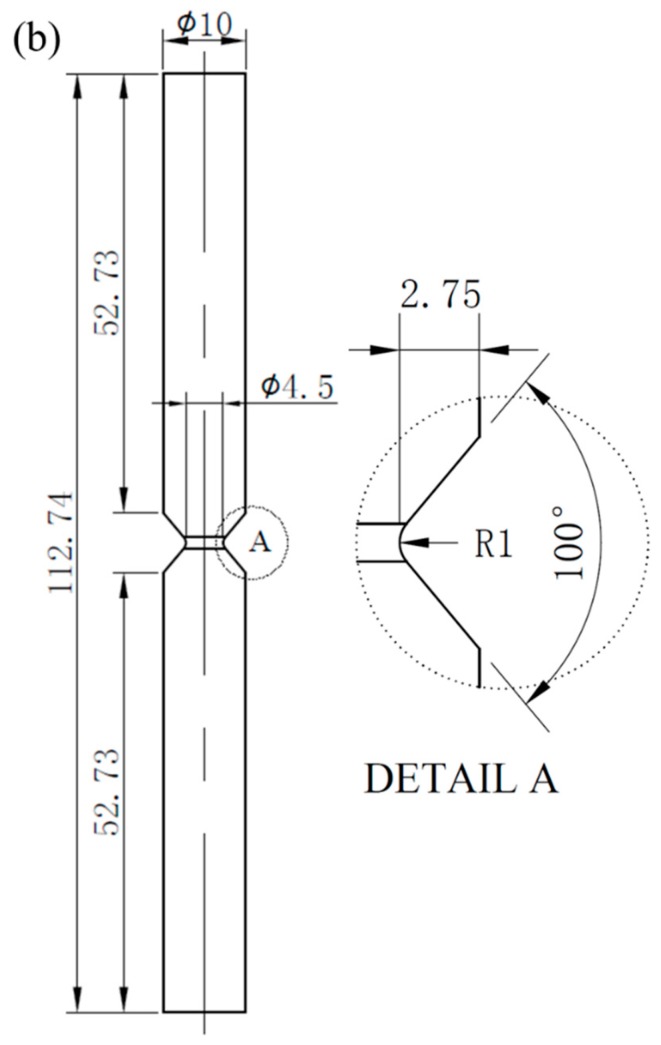
Shapes and dimensions (units: mm) of (**a**) smooth specimen and (**b**) notched specimen for ultrasonic fatigue tests; notch radius (R) is 1 mm.

A self-developed ultrasonic fatigue test device was utilized to perform fatigue experiments at various stress ratios. By attaching an ultrasonic fatigue test machine to a conventional uniaxial tensile test machine (Shimadzu AG-X *plus*), a constant stress is superimposed on the ultrasonic cyclic load. Thus, the variable stress ratio loading was achieved. In order to improve experimental efficiency, a commercial ultrasonic fatigue test machine (USF-2000, Shimadzu, Kyoto, Japan) was used to conduct fatigue tests at *R* = −1. During fatigue tests, the compressed dry air was used to cool the middle part of specimens. Figure 2 shows the geometries of two kinds of specimens for ultrasonic fatigue experiments. The specimens were designed to work at a resonance frequency of 20 kHz with ultrasonic fatigue machines. The screw was machined at just one end of the specimens at *R* = −1, while both two ends of the specimens had screws at *R* > −1. All specimens in this study were machined from the as-received bars with a diameter of 25 mm. Mechanical mirror polishing was performed on the surface of the diameter reduced portion to eliminate scratches. For smooth specimens, only fatigue property at *R* = −1 was investigated, while three stress ratios of −1, 0.1 and 0.5 were chosen to study the fatigue properties of the notched specimens. Moreover, the elastic stress concentration factor (*K_t_*) of the notched specimen was calculated as 1.82 by finite element method.

After the fatigue tests, all fracture surfaces of the failure specimens were examined by a scanning electron microscope (SEM, JSM-6510LV, JEOL, Tokyo, Japan). The projective dimensions of the crack initiation regions were measured from the SEM photographs. Furthermore, the tensile test of notched specimen was also conducted.

## 3. Experimental Results

Although notch geometry decreases the fatigue properties of materials [22,23,24], it is capable of enhancing tensile strength [25]. Before we investigated the fatigue properties of this material, the nominal tensile strength of 1353 MPa for the notched specimen shown in Figure 2b was determined. The tensile strength of the notched specimen increased 45.8% over the smooth specimen (928 MPa). The reduction of area for the notched specimen was lower than the smooth one [25], which can be attributed to the stress confinement effect induced by the notched configuration. It resulted in higher strength and lower ductility for the notched specimen [25].

**Figure 3:**
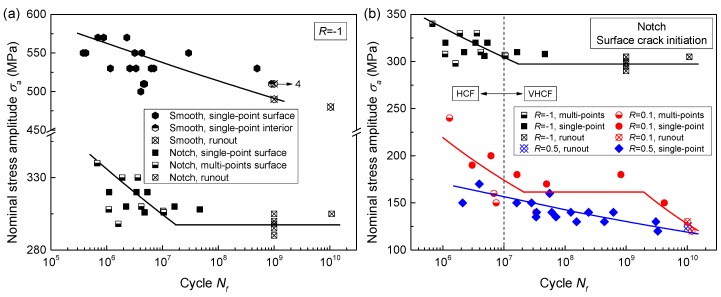
(**a**) *S*-*N* data and curves for the smooth specimen and the notched specimen at *R* = −1, and (**b**) *S*-*N* data and curves for the notched specimen at *R* = −1, 0.1 and 0.5. The nominal stress amplitude was obtained by dividing the applied force by the minimum cross-sectional area. (Single-point: crack initiation with single-point; multi-points: crack initiation with multi-points; runout: no broken; the numeral at the right side of the arrow indicates the quantity of same test results.).

Figure 3 presents the results of *S*-*N* data and curves for the smooth specimen at *R* = −1 and for the notched specimen at *R* = −1, 0.1 and 0.5. The testing fatigue life varied from 10^5^ cycles to 10^10^ cycles as shown in Figure 3. A three-parameter equation was employed to calculate the stress plateau values of *S*-*N* curves. Its formula is expressed as:

(1) $${\left(S-S_{f}\right)}^{m}\cdot N_{f}=c,$$

where *S* is the applied stress level, which refers to nominal stress amplitude (*σ_a_*) in this study. *S_f_* is fatigue limit, that is, the stress plateau value. *N_f_* is fatigue life. *m* and *c* are two constants. As a result, the stress plateau values of 297.4 MPa for the notched specimens at *R* = −1 and 161.5 MPa for the notched specimens at *R* = 0.1 were numerically obtained by nonlinear fitting with Equation (1). In continuous declining regions of *S*-*N* data, their *S*-*N* curves were statistically determined for a 50% survival probability. The well-known Basquin equation was used at this moment. According to the above methods, all *S*-*N* curves in the whole life region were obtained, as presented in Figure 3. Note that the unbroken specimens were treated as failure in this study, so the results are conservative.

As seen in Figure 3a, the notch geometry made *S*-*N* curves change from a continuous declining shape to a horizontal asymptote shape. For the notched specimens, the *S*-*N* curves at three stress ratios presented three different shapes, as shown in Figure 3b, which can be explained by the competition between fatigue failure and cyclic creep failure [26]. Based on the calculated *S*-*N* curves, the fatigue strength at a specific fatigue life was determined and summarized in Table 1.

On the basis of the quantity and location of crack initiation point, all fracture surfaces can be divided into three types, i.e., single-point interior crack initiation, single-point surface crack initiation and multi-point surface crack initiation. As shown in Figure 3, only one smooth specimen failed with single-point interior crack initiation at about 10^9^ cycles. All the fracture surfaces of notched specimens presented surface crack initiation.

**Figure 4:**
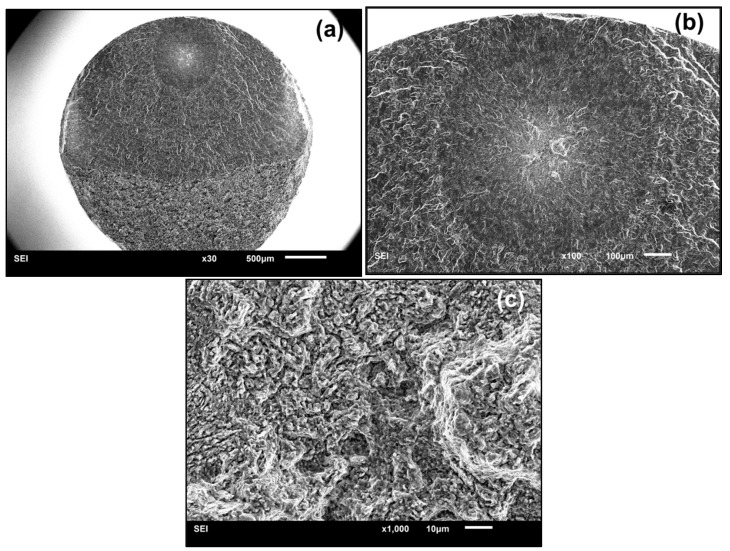
Typical fractography of single-point interior crack initiation without facet feature (smooth specimen, *R* = −1, *σ_a_* = 510 MPa, *N_f_* = 9.0692 × 10^8^ cycles); (**a**) the whole fracture surface; (**b**) enlargement of the “fish-eye”; (**c**) enlargement of the crack initiation region in the center of the “fish-eye”.

Figure 4 shows the fractography of single-point interior crack initiation. It presented the “fish-eye” failure mode, which is frequently observed in high-strength steels [2,4,27]. However, a large number of fine granules, rather than inclusions, appeared in the center of the “fish-eye”, as shown in Figure 4c. For titanium alloys, similar morphology was found at *R* = −1 in other literatures [10,15]. With increase in the applied cyclic loading, the micro-cracks first form in α_p_ grains, then coalesce to micro-crack clusters with extremely rough paths, which finally results in interior crack initiation failure in the VHCF regime [15]. During the coalescence processes in the interior of α_p_ grains and between neighboring grains, numerous cyclic pressing results in grain refinement of the interior crack surface, which accounts for the formation of fine granules [11,15].

**Figure 5:**
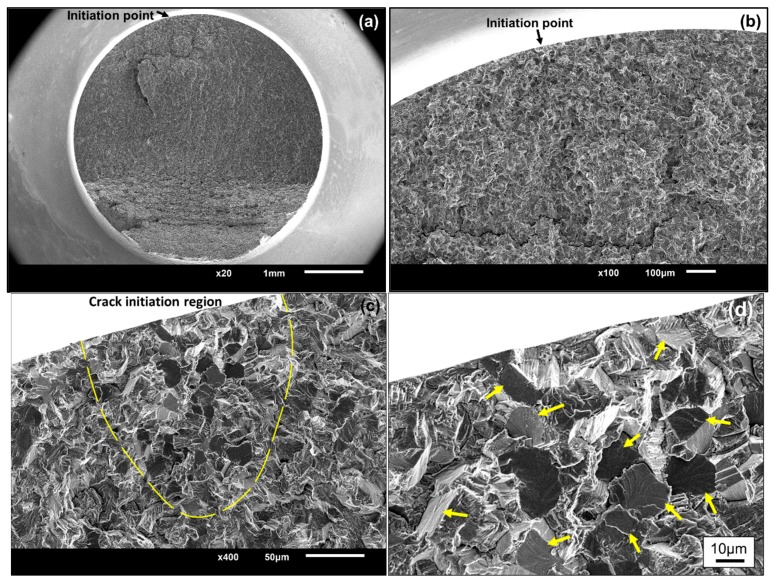
Typical fractography of single-point surface crack initiation with clear facet feature (notched specimen, *R* = 0.5, *σ_a_* = 140 MPa, *N_f_* = 6.048 × 10^7^ cycles); (**a**) the whole fracture surface; (**b**) macro profile with crack initiation region and crack propagation region; (**c**) enlargement of crack initiation region recognized by the facet feature and separated by the dashed line; (**d**) enlargement of the facets, as pointed out by the arrows.

**Figure 6:**
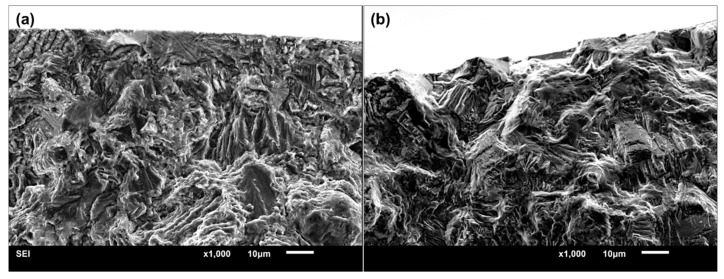
Typical fractography of single-point surface crack initiation region with blurry facet feature at *R* = −1, (**a**) smooth specimen (*σ_a_* = 530 MPa, *N_f_* = 2.5939 × 10^6^ cycles) and (**b**) notched specimen (*σ_a_* = 320 MPa, *N_f_* = 3.4223 × 10^6^ cycles).

**Figure 7:**
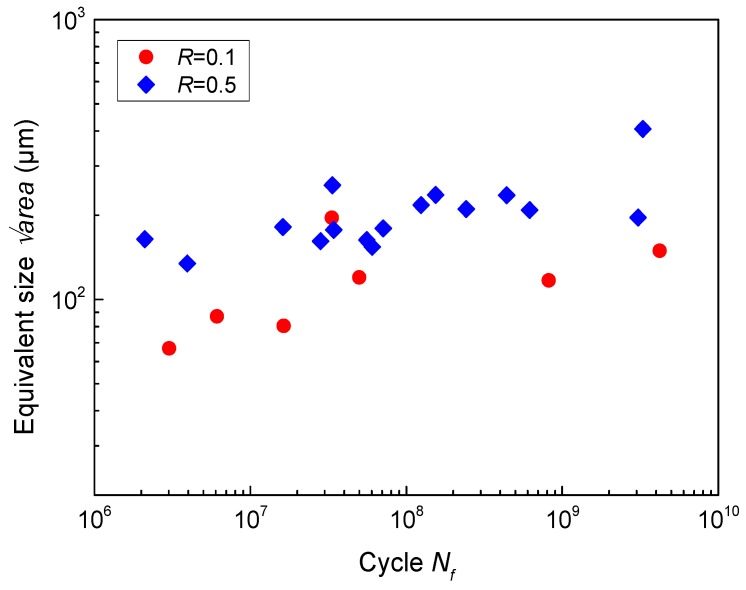
The equivalent size of the single-point surface crack initiation region versus fatigue life for notched specimens.

The SEM photographs shown in Figure 5 are an example of the typical fractography of single-point surface crack initiation. For titanium alloys, the facet morphology is discovered frequently in the crack origin region, especially in positive stress ratios [7,8,10,28]. A large number of studies [8,17,26,29,30] have shown that the formation of facets results from the planar slips or cleavages of α_p_ grains, as shown in Figure 1. The critical microstructure resulting in crack initiation is the preferred micro-texture of α_p_ grains for this alloy [26]. The formation of facets and their coalescence process constitute the whole crack initiation stage [8,19]. Thus, by utilizing the facets presented in Figure 5d, the crack initiation region can be recognized completely by the dashed line, as depicted in Figure 5c. The shape of the dashed line totally depends on the distribution of facets; they were of irregular shape in most cases. The projected area normal to the applied stress direction of the single-point surface crack initiation region (*area*) was measured by this method from their SEM photographs. It should be noted that the facet feature in crack initiation region is blurry at *R* = −1, as shown in Figure 6. The compression and friction among upper and lower crack surfaces possibly lead to the occurrence of this morphology when pressure is applied. Hence, to ensure accuracy of measurement, only the values of *area* at positive stress ratios were measured. Finally, the equivalent sizes ( $\sqrt{area}$), introduced by Murakami [31], were determined by calculating the square root of the values of *area* and plotted in Figure 7. Obviously, the equivalent sizes increased with the increase of fatigue life and stress ratio. Similar results were reported by others [6]. For surface crack initiation, the stress intensity factor range (Δ*K*) at crack tip can be calculated by [31]

(2) $$\Delta K=0.65\cdot \Delta \sigma \cdot \sqrt{\pi \sqrt{area}}$$

Because the Δ*K* at the tip of the crack initiation region corresponds to the threshold value of fatigue crack propagation. Higher fatigue life and stress ratio means lower applied stress amplitude (Δ*σ*), as shown in Figure 3b. Thus, in order to remain constant for the threshold value of fatigue crack propagation [6], the equivalent sizes increased with the increase of fatigue life and stress ratio, according to Equation (2).

**Figure 8:**
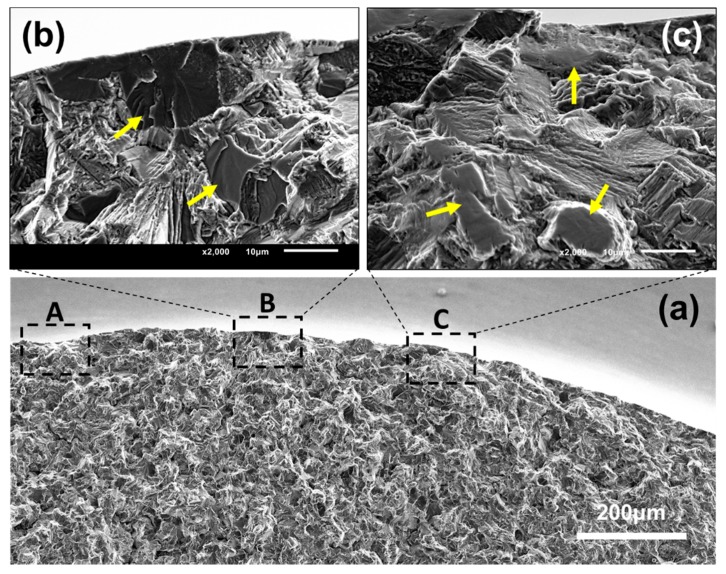
Typical fractography of multi-point crack initiation with three origin sites of A, B and C (notched specimen, *R* = 0.1, *σ_a_* = 150 MPa, *N_f_* = 7.460 × 10^6^ cycles); (**a**) the macro profile with three crack initiation regions and crack propagation region; (**b**) enlargement of crack initiation region B; (**c**) enlargement of crack initiation region C.

Another typical fractography of multi-point surface crack initiation with three origin sites is presented in Figure 8. The obvious radial ridges, formed during crack propagation, were observed in the vicinity of all three origin sites. This indicates that all three origin sites belong to effective crack initiation points. That is, after these micro-cracks initiate, they grow together and subsequently coalesce with each other. As seen in Figure 3, the multi-point crack initiation behavior was not detected for the smooth specimens, while it prevailed in the notched specimens at *R* = −1. Meanwhile, with the increase of stress ratio, the number of specimens with multi-point surface crack initiation decreased rapidly. Almost all of the multi-point surface crack initiation failure appeared in the HCF regime (<10^7^ cycles). Similar to the morphology of the single-point surface crack initiation region, facets were detected in the crack initiation region of this failure mode, as shown in Figure 8b,c.

## 4. Discussion

### 4.1. Multi-Point Crack Initiation Effect on Fatigue Properties

When *R* = −1, the true stress range at the surface rim region, calculated by multiplying the nominal stress amplitude by the stress concentration factor, from 10^5^ to 10^7^ cycles, is 674.7–554.2 MPa for the notched specimen, which is significantly greater than the true stress range of 588.0–537.5 MPa for the smooth specimen, especially in relative low fatigue life. Both higher loading stress at the same stress ratio [21] and lower applied stress ratio [26] will cause fatigue crack initiation with multi-points. Thus, the multi-point crack initiation behavior prevailed in notched specimen at *R* = −1, while this feature was not detected in the smooth specimen at *R* = −1 and the notched specimen at *R* = 0.5, as shown in Figure 3. With increase in the fatigue life, the transition of fatigue failure mode from multi-point crack initiation to single-point crack initiation will occur [14]. This feature is consistent with the results presented in Figure 3b. Almost all specimens with multi-point crack initiation failed in the low fatigue life regime (HCF). Therefore, only HCF properties are affected by multi-point crack initiation behavior.

As seen in Figure 8, the distance among these crack origin sites is about 350 μm. That is to say, their locations are very close. A similar feature was widely detected in other multi-point crack initiation behaviors in this study. On one hand, the stress concentration induced by original micro-crack will result in the existence of a high stress region around the micro-crack. This high stress region can promote the initiation process of an adjacent original micro-crack. Moreover, these adjacent micro-cracks influence each other. Namely, multi-point crack initiation can reduce crack initiation life. On the other hand, when the size of an original micro-crack reaches the critical value of crack propagation, these micro-cracks will subsequently coalesce with each other into a macroscopic crack after growth independence in a very short distance. The coalescence process can decrease crack propagation life. In summary, the multi-point crack initiation behavior can decrease crack initiation life and crack propagation life, that is, the total fatigue life. Therefore, this failure mode frequently appeared in the left side of the *S*-*N* curves, as depicted in Figure 3. Meanwhile, the coexistence of single-point and multi-point crack initiation at the same stress level increased the scatter of fatigue life data.

When fatigue life is lower than 10^6^ cycles, the crack propagation life occupies a large proportion of total life [10,32]. However, when fatigue life varies from 10^6^ to 10^7^ cycles, most of the total life is consumed in the crack initiation process, especially in 10^7^ cycles (over 95%) [10,32,33]. Based on the above discussion, it can be concluded that when the total life is lower than 10^6^ cycles, the fatigue life reduced by multi-point surface crack initiation mainly results from the crack propagation stage. However, the reduced fatigue life mainly occurs in the crack initiation process, when the total life varies between 10^6^ cycles and 10^7^ cycles.

### 4.2. Mean Stress Effect on Fatigue Properties

**Figure 9:**
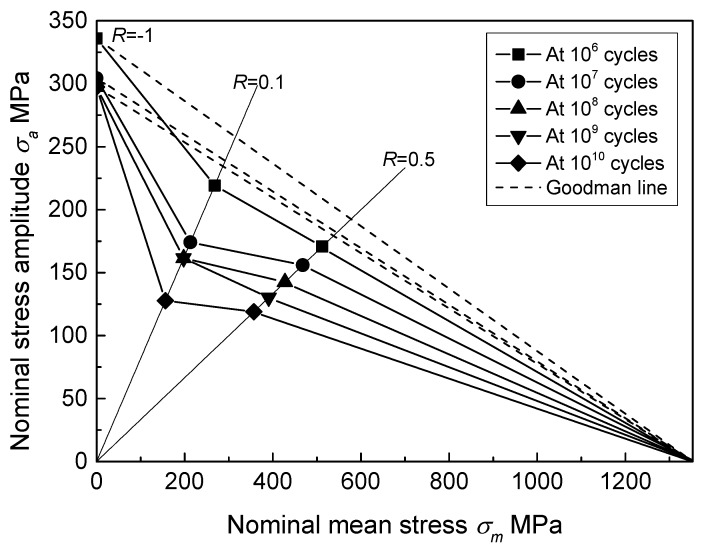
Haigh diagram of the notched specimen at different fatigue life.

The Haigh diagram is usually utilized to represent the effect of mean stress on fatigue property [34]. It presents the relationship between mean stress (*σ_m_*) and stress amplitude (*σ_a_*) at a given fatigue life. On the basis of the fatigue strength data listed in Table 1, the Haigh diagram for the notched specimen in HCF and VHCF regimes is plotted in Figure 9. Goodman approximations are also shown by the dashed lines for comparison, which present a linear relationship between the fatigue strength expressed by stress amplitude at *R* = −1 (*σ_a_*_,*R*=−1_) and ultimate tensile strength (*σ_UTS_*):

(3) $$\sigma_{a}=\sigma_{a,R=-1}\cdot \left(1-\frac{\sigma_{m}}{\sigma_{UTS}}\right)$$

where the nominal stress is adopted and the ultimate tensile strength of notched specimen is employed, owing to the notch effect.

In general, Goodman approximation provides a conservative estimation for structural materials [35]. Thus, this method is widely used to estimate the mean stress effect in fatigue design of structural parts. However, all fatigue strength data at *R* = 0.1 and 0.5 passed through the conservative Goodman lines into the dangerous side, as shown in Figure 9. With fatigue life increased, the deviation from Goodman lines became more distinct. It demonstrates that more obvious degeneration of fatigue strength at *R* > −1 is detected in the VHCF regime than in the HCF regime. For titanium alloys, similar degeneration trend was reported, but the degree of degeneration is weaker for smooth specimens [5,7,10]. In particular, when fatigue life is lower than 10^7^ cycles (HCF), the Goodman line still provides estimation with a good margin of safety for the smooth specimen [5,10]. However, the Goodman approximation could no longer be applied for notched specimens in the HCF regime, as shown in Figure 9. This indicates that notch effect further promotes the reduction of fatigue strength at *R* > −1 in the HCF and VHCF regimes.

**Figure 10:**
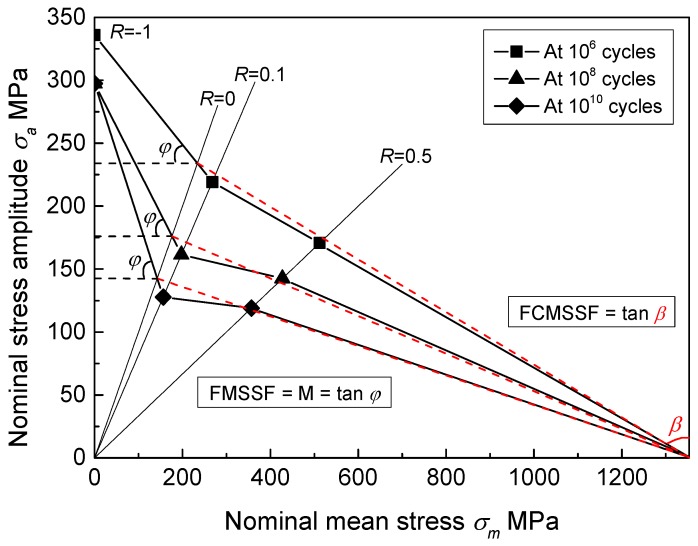
Schematic representation of FMSSF and FCMSSF.

To quantify the mean stress sensitivity of fatigue strength, Mayer [36] proposed a linear method for spring steel. According to Figure 9, a linear relationship is not suitable for calculating mean stress sensitivity for this titanium alloy. Therefore, based on the results in Figure 9 and Goodman method, a bilinear model was proposed. With the increase of stress ratio from −1 to 1, the dominance of fatigue on specimen failure is weaker, while the effect of cyclic creep on specimen failure gets stronger [7,26,37]. At the surface of notched specimens, the true value of applied maximum stress at *R* = 0.5 exceeded 873.6 MPa by linear elastic theory, which is slightly greater than the yield strength of 820 MPa. This demonstrates that plastic deformation occurred at the surface of notched specimens at *R* = 0.5; thus, the cyclic creep effect must be taken into account at high stress ratios. Based on the above discussion, the mid-value of *R* = 0 (*σ_a_* = *σ_m_*) was chosen as the inflection point of the bilinear model, as illustrated in Figure 10. When stress ratio varies from −1 to 0, the fatigue mean stress sensitivity factor (FMSSF) is defined as:

(4) $$\mathrm{FMSSF}=\tan(\varphi )=\frac{\sigma_{a,R=-1}-\sigma_{a,R=0}}{\sigma_{m,R=0}}=\frac{\sigma_{a,R=-1}}{\sigma_{m,R=0}}-1$$

where *σ_a_*_,*R* = 0_ and *σ_m_*_,*R* = 0_ are stress amplitude and mean stress at *R* = 0, respectively. The definition of FMSSF is completely consistent with the mean stress sensitivity factor (M) proposed by Schütz [38]. When stress ratio changes from 0 to 1, the fatigue-creep mean stress sensitivity factor (FCMSSF) is defined as:

(5) $$\mathrm{FCMSSF}=\tan(\beta )=\frac{\sigma_{UTS}-\sigma_{m,R=0}}{\sigma_{a,R=0}}=\frac{\sigma_{UTS}}{\sigma_{a,R=0}}-1$$

This factor is a newly developed concept. These two factors took the stress ratio from −1 to 1 into account and showed a good match with the test results. As a result, the calculated values of these two factors are listed in Table 2.

The values of FMSSF and FCMSSF increased with the increase of fatigue life. Namely, the fatigue mean stress sensitivity and fatigue-creep mean stress sensitivity of this material correlate with the fatigue life for the notched specimen. The increase of fatigue life from the HCF to VHCF regime will enhance these two sensitivities. The values of two sensitivity factors in 10^10^ cycles were significantly greater than the values in 10^7^ cycles, which correspond to the traditional fatigue limit. That is to say, for fatigue design with ultra-long life, a significantly higher safety margin must be adopted than traditional fatigue design.

### 4.3. Estimation of Fatigue Strength

Few models are able to correlate the macro-mechanical properties and microstructure features of fracture surfaces. Based on a large number of experimental data, Murakami [31] summarized an empirical formula for calculating fatigue limit (*σ_w_*) of materials containing small defects or inclusions at *R* = −1. For surface crack initiation, the formula is expressed by:

(6) $$\sigma_{w}=1.43\left(HV+120\right)/{\left(\sqrt{area}\right)}^{1/6}$$

where *HV* is the Vickers hardness of the materials. $\sqrt{area}$ refers to the square root of the projected area of an inclusion or small defect perpendicular to the loading direction. The nature of the model is that there is critical stress for an inclusion or small defect. When the applied fatigue stress level is lower than the critical stress, the existing micro-crack (inclusion or small defect) does not propagate. From a fracture mechanics perspective, the stress intensity factor range at the front of the micro-crack is equal to the threshold stress intensity factor range for crack propagation when critical stress is reached. Critical stress is termed as fatigue limit corresponding to the equivalent size of $\sqrt{area}$. This model has been widely used for materials containing inclusions [24,27,37]. However, little effort has been made on extending the model to materials without inclusions or small defects, such as titanium alloys. This may be because the facets (rather than inclusions) exist in the crack initiation region for titanium alloys, as shown in Figure 5 and Figure 8. It is more difficult to quantificationally measure equivalent size than inclusions.

The stress intensity factor range at the front of the crack initiation region recognized by facets is equal to the threshold for crack propagation for titanium alloys [6,10]. Namely, the equivalent sizes in Figure 7 possess the same nature with $\sqrt{area}$ in Equation (6). Thus, it is reasonable to substitute the sizes in Figure 7 into the model. For this alloy, fatigue failure occurred over 10^7^ cycles as shown in Figure 3; no fatigue limit exists. Therefore, fatigue strength, rather than fatigue limit, should be discussed.

Based on Equation (6), a modified factor was proposed empirically by Murakami to take stress ratio into account. When *R* = −1, this modified model should reduce to Equation (6). Finally, the model is presented by the following equation:

(7) $$\sigma_{w}=\frac{1.43(HV+120)}{{\left(\sqrt{area}\right)}^{1/6}}\cdot {\left(\frac{1-R}{2}\right)}^{\alpha }$$

where

(8) $$\alpha =0.226+HV\times 10^{-4}$$

However, this model does not consider the effect of stress concentration induced by notch geometry on fatigue strength. Similar to the modified factor of stress ratio, a new factor was proposed to modify the effect of stress concentration. When *K_t_* = 1, the new model should reduce to Equation (7). Therefore, the following possible equation may be assumed:

(9) $$\sigma_{w}=\frac{1.43(HV+120)}{{\left(\sqrt{area}\right)}^{1/6}}\cdot {\left(\frac{1-R}{2}\right)}^{\alpha }\cdot {\left(\frac{2}{1+K_{t}}\right)}^{\delta }$$

where *δ* is a coefficient in connection with materials. Substituting the equivalent sizes at *R* = 0.1 in Figure 7 and the corresponding fatigue strength into Equation (9), the mean value of *δ* was determined as 0.908 for this alloy. From now on, Equation (9) can be used to estimate the fatigue strength of each specimen at *R* = 0.5. The final results calculated by Equations (7) and (9) are listed in Table 3 for comparison.

The errors in Table 3 are obtained by the following formula:

(10) $$\mathrm{Err}.=\left(\sigma_{w}-\sigma_{a}\right)/\sigma_{a}$$

As a result, after introducing the modified factor of stress concentration, the accuracy of estimated values improved significantly. The new model gave a maximum error of 15.9% from 10^6^ to 10^9^ cycles. Meanwhile, 87.5% estimated results agreed to within 10%. In conclusion, the new model based on the Murakami model can provide more appropriate predictions for notch fatigue strength by taking stress concentration into account in the HCF and VHCF regimes.

## 5. Conclusions

The present investigation on the effects of notch and mean stress on HCF and VHCF properties of a near-alpha titanium alloy draws the following conclusions:

(1) Multi-point crack initiation behavior only prevailed in the notched specimens at *R* = −1. Almost all of the multi-point crack initiation failure appeared in the HCF regime. With increase in the total life in the HCF regime, the source of reduced fatigue life caused by multi-point crack initiation changes from crack propagation stage to crack initiation stage.

(2) More obvious degeneration of fatigue strength at *R* > −1 is detected in the VHCF regime than the HCF regime. Notch effect further promotes the reduction of fatigue strength at *R* > −1 in the HCF and VHCF regimes.

(3) The bilinear model, extended from the Goodman method, can better estimate the mean stress sensitivity of this titanium alloy. The fatigue mean stress sensitivity and fatigue-creep mean stress sensitivity increased with the increase of fatigue life in the HCF and VHCF regimes.

(4) The new model, based on the Murakami model, can provide more appropriate predictions for notch fatigue strength by taking stress concentration into account in the HCF and VHCF regimes.

## Figures and Tables

**Table 1:** Fatigue strength at different number of loading cycles.

Specimen Shape	Stress Ratio	10^6^	10^7^	10^8^	10^9^	10^10^
Smooth	−1	562.2	537.5	513.9	491.4	-
Notch	−1	336.0	304.5	297.4	297.4	297.4
	0.1	219.2	174.1	161.5	161.5	127.7
	0.5	170.7	156.0	142.5	130.2	119.0

**Table 2:** Values of FMSSF and FCMSSF at different fatigue life.

Fatigue Life	10^6^	10^7^	10^8^	10^9^	10^10^
FMSSF	0.4357	0.6129	0.6885	0.6885	1.0868
FCMSSF	4.7817	6.1663	6.6817	6.6817	8.4937

**Table 3:** A comparison of the estimated values of fatigue strength calculated by Equations (7) and (9) together with experimental results.

Specimen Code	*N_f_*	$\sqrt{area}\;(\mu m)$	*σ_a_* (MPa)	*σ_w_* [Equation (7)] (MPa)	*σ_w_* [Equation (9)] (MPa)	Err. [Equation (7)] (%)	Err. [Equation (9)] (%)
9	2.106 × 10^6^	164.0	150	189	138	26.0	−7.8
1	3.940 × 10^6^	134.4	170	195	143	14.9	−15.9
16	1.618 × 10^7^	181.4	150	186	136	23.9	−9.3
3	2.820 × 10^7^	161.5	150	189	139	26.3	−7.6
11	3.358 × 10^7^	256.0	135	175	128	30.0	−4.9
6	3.424 × 10^7^	177.2	140	187	137	33.2	−2.5
2	5.579 × 10^7^	163.1	160	189	138	18.2	−13.5
7	6.048 × 10^7^	154.0	140	191	140	36.4	−0.2
12	7.114 × 10^7^	179.3	135	186	136	37.9	0.9
5	1.244 × 10^8^	217.4	140	180	132	28.8	−5.7
13	1.543 × 10^8^	236.3	130	178	130	36.8	0.1
10	2.423 × 10^8^	210.3	140	181	133	29.5	−5.2
17	4.401 × 10^8^	235.5	130	178	130	36.8	0.2
4	6.177 ×10^8^	208.6	140	182	133	29.7	−5.1
8	3.062 × 10^9^	196.1	130	183	134	41.1	3.3
14	3.289 × 10^9^	406.7	120	162	119	35.3	−0.9
